# P04.52. Alternative and complementary health practices among college students: implications for health promotion in higher education

**DOI:** 10.1186/1472-6882-12-S1-P322

**Published:** 2012-06-12

**Authors:** A Burke

**Affiliations:** 1 San Francisco State University, San Francisco, USA

## Purpose

Alternative and Complementary Health Practices (ACHP) may provide new opportunities for encouraging positive health behaviors among college students, such as reducing excessive alcohol use, a persistent campus challenge. To explore this issue a survey was conducted to examine the relationship between ACHP use and health behaviors.

## Methods

The CORE, a national college drug and alcohol survey, was completed by a convenience sample of 2,312 respondents at a state university. The 39-item survey was augmented with 18 questions, including items on ACHP use and identification with postmodern social values.

## Results

For comparison respondents were dichotomized into ACHP users and non-users – based on their response to a frequency of use survey item. This produced a group of 531 (31.3%) ACHP-users and 1,164 non-users. ACHP students were significantly more likely to be female (71.4% users versus 63.0% non-users, p=.001) and older (24.4 versus 23.3, p=.001). There were no statistically significant differences between the two groups in terms of past 30 day use of alcohol, tobacco, or marijuana, binge drinking, or perceived risks from binge drinking. ACHP students were significantly more likely to report engaging in conventional health practices (p=.001), and having a higher self-reported health status (p=.007). They also reported experiencing more mental health symptoms during the school year (p=.012). Finally, ACHP students were significantly more likely to report identification with postmodern social values including interest in the environment (p=.001) and social justice (p=.001), and to having a somewhat more liberal political orientation.

## Conclusion

Despite a lack of significant differences in alcohol use and other risk behaviors, there is a foreseeable long-term value in promoting ACHP on college campuses due to its current use by many students (31.3% reporting use in the past 30 days), its relationships with other conventional preventive health practices, and its association with positive social values.

